# Study on the Microscale Mechanisms of Multi-Source Solid Waste Synergy in Enhancing Physicochemical Properties of Red Mud-Based Backfill Materials

**DOI:** 10.3390/ma18081822

**Published:** 2025-04-16

**Authors:** Jinjing Huang, Guochao Yan, Shaoqi Kong, Xuyang Bai, Jiawei Zhang, Zhiguo Ge

**Affiliations:** 1 School of Mining Engineering, Taiyuan University of Technology, Taiyuan 030024, China; huangjinjing0805@link.tyut.edu.cn (J.H.);; 2 School of Energy and Mining Engineering, China University of Mining and Technology (Beijing), Beijing 100083, China; bqt2400101035@student.cumtb.edu.cn

**Keywords:** red mud, industrial solid waste, synergistic effect, backfill material, resource utilization

## Abstract

To address the issues of the low pozzolanic activity and high pollution potential of red mud (RM), this study utilizes different industrial solid wastes to synergistically enhance the physicochemical properties of red mud-based filling materials. The compressive strengths of red mud-based filling materials activated by three types of solid wastes—desulfurized gypsum (DG), carbide slag (CS), and steel slag (SS)—were compared, revealing the differences in their effects on the physicochemical properties of the materials. The results showed that DG significantly enhanced the compressive strength of the backfill material. The composite system composed of 65.8% RM, 18.8% FA, 9.4% cement, and 6% DG achieved a compressive strength of 7.36 MPa after 28 days of curing, demonstrating a 97.8% increase compared to the control group. Techniques such as X-ray diffraction (XRD), Fourier-transform infrared spectroscopy (FTIR), scanning electron microscopy (SEM), and Brunauer–Emmett–Teller (BET) analysis were employed to characterize the microstructural evolution of the red mud-based filling materials activated by different solid wastes. This study investigated the differences in the pore structure, microscopic morphology, and chemical composition of the materials containing different solid wastes. The results indicated that DG effectively promotes the formation of ettringite and C(-A)-S-H gel, optimizes the pore structure of the filling materials, and forms a dense matrix, thereby enhancing the stiffness and strength of the materials. Additionally, the red mud-based filling materials developed in this study exhibit excellent environmental performance. This not only provides theoretical support for the development of red mud-based filling materials but also offers new insights for mine backfilling and the co-disposal of solid wastes.

## 1. Introduction

The generation of industrial solid waste in China continues to rise [1,2], with bulk industrial solid waste reaching 4.038 billion tons in 2021 and there being a comprehensive utilization rate of 57.56%. However, the utilization rate of red mud is only 6.68%, indicating low efficiency. Red mud is a byproduct of aluminum industrial production [3,4]. Depending on the quality of bauxite and the characteristics of the process, 1.0 to 1.8 tons of red mud is generated for every ton of alumina produced [5,6]. Due to its high alkalinity, complex chemical composition, and toxic heavy metals such as arsenic, lead, and mercury, red mud is difficult to dispose of. Most alumina plants in China and elsewhere transport red mud to storage yards for accumulation [7,8]. However, long-term storage can lead to soil alkalization, the destruction of microbial communities, and the leaching of alkaline substances and heavy metals into water bodies during heavy rainfall, causing elevated pH levels and ecological toxicity. Therefore, how to effectively dispose of and utilize red mud has become an unavoidable issue in the development of the aluminum industry [9,10,11].

Research shows that red mud utilization primarily focuses on three fields: the construction and chemical industry [12,13,14,15,16], the environmental protection and agriculture industry [17,18,19,20,21], and the valuable element extraction industry [22,23,24]. In the construction and chemical materials industry, red mud has been successfully applied in manufacturing concrete, catalysts, composite materials, adsorbents, and geopolymers. Environmental/agricultural research focuses on its use in water/waste gas treatment and soil improvement, while the valuable element extraction field targets the recovery of metals such as iron, aluminum, and titanium from red mud. Although environmental/agricultural and extraction applications have achieved partial economic benefits, their industrial-scale implementation remains constrained by technical bottlenecks, hindering large-scale red mud consumption. In contrast, converting red mud into construction/chemical materials currently stands as the most effective method for bulk utilization.

In the field of construction and chemical materials, researchers have utilized red mud to produce cement-based materials (concrete admixtures, geopolymers), catalytic materials, and composite materials [25,26,27,28]. Particular attention has been paid to using red mud-based cementitious materials for mine backfilling to address mining-induced geological hazards such as ground subsidence, fissures, collapses, landslides, and debris flows in mined-out areas [29,30,31,32]. Some scholars have prepared cement-based materials by replacing part of the clay in cement and concrete with red mud or using red mud as concrete aggregate. For instance, Yao et al. [33] employed red mud and coal industry byproducts as raw materials for cement-based material production, demonstrating that the resulting red mud–coal byproduct cementitious material meets ASTM standards for physical and mechanical properties. Liu et al. [34] developed concrete using Bayer process red mud, finding that its strength activity index resembled that of fly ash. With red mud incorporation, the elastic modulus, compressive strength, and splitting tensile strength of concrete increased, while shrinkage decreased. However, such applications require the strict control of red mud dosage or dealkalization pretreatment. Additionally, the insufficient activation of red mud’s reactivity limits its broader utilization in these cement-based systems.

Therefore, researchers have begun to focus on using red mud to prepare geopolymers, enhancing their pozzolanic activity through mechanical activation and the addition of chemical reagents such as sodium silicate to produce geopolymers with excellent performance. This method not only effectively activates the pozzolanic activity of red mud, transforming it into a cementitious material, but also results in geopolymers with superior mechanical properties and stable physical and chemical performance while avoiding pollution issues during the production process. Ye et al. [35] synthesized a single-component geopolymer using alkali-thermal activated Bayer red mud as the raw material and adding silica to optimize its composition. Wang et al. [36] used Bayer red mud, fly ash, and cement as raw materials and employed an orthogonal experimental method to evaluate the strength performance of Bayer red mud in cement paste backfill, determining the optimal mix ratio for the paste. Li et al. [37] prepared geopolymers using Bayer red mud and fly ash, finding that mechanical activation improved the activity of aluminosilicate in the gelated red mud, thereby effectively promoting the reaction between fly ash and red mud. When 14% sodium silicate was added to the binder, the compressive strength of the samples was further enhanced. However, preparing red mud geopolymers through mechanical activation and the addition of chemical reagents still requires extra active aluminosilicates to improve the strength of the geopolymers, resulting in higher costs for the red mud geopolymers produced by this method, making it difficult to use them as filling materials for goaves.

Current technologies face two core challenges: First, the low pozzolanic activity of red mud necessitates reliance on high-cost dealkalization or external silicon–aluminum activators to enhance its cementitious properties. Second, traditional activators (e.g., sodium silicate) improve reactivity but substantially increase material costs, limiting their application in large-scale projects such as mine backfilling. To address these issues, this study proposes an innovative strategy utilizing multi-component industrial solid wastes to synergistically activate red mud reactivity, achieving collaborative performance enhancement in red mud-based backfill materials. A “solid waste–red mud” multiphase reaction system will replace costly chemical reagents, developing mechanically robust and environmentally compatible materials for mine void backfilling.

Specifically, three industrial solid wastes (DG, CS, SS) will be employed to synergistically activate red mud’s pozzolanic activity for producing backfill materials with balanced mechanical and environmental performance. Macroscopic analyses, including uniaxial compression tests and energy evolution patterns, will reveal the mechanical reinforcement mechanisms of different solid wastes. Advanced mesoscale characterization techniques (XRD, SEM, BET, FTIR), combined with mechanical test results, will be used to investigate phase composition, microstructural evolution, and hydration process enhancement mechanisms. This integrated approach aims to elucidate the multiscale effects of solid wastes on material performance and structural development. This study will establish a theoretical foundation and technical framework for developing red mud-based backfill materials suitable for mine voids, ultimately seeking to promote resource recycling and environmental sustainability.

## 2. Materials and Methods

### 2.1. Materials

In this experimental study, six materials were selected: red mud (RM), fly ash (FA), desulfurized gypsum (DG), carbide slag (CS), steel slag (SS), and Portland cement (PC). Their chemical compositions and mineral phase structures were analyzed using X-ray fluorescence (XRF) spectroscopy and X-ray diffraction (XRD) techniques. The machine model used for XRF analysis in this test is panalytical Axios. The machine model used for XRD analysis in this test is panalytical empyrean in the Netherlands.

Specifically, RM was sourced from the An aluminum plant in Shanxi. Its chemical composition primarily consisted of Fe_2_O_3_, Al_2_O_3_, SiO_2_, Na_2_O, and TiO_2_, which collectively accounted for 97.83% of the total mass fraction. FA was obtained from a large power plant in Shanxi Province. The main chemical components included Fe_2_O_3_, Al_2_O_3_, SiO_2_, CaO, and SO_3_, with a combined mass fraction of 95.72%. DG was provided by a large power plant in Shanxi Province. Its chemical composition was dominated by CaO and SO_3_, which together constituted 99.83% of the total, with trace components accounting for less than 0.2%. CS was collected from a chemical plant in Shanxi Province. The primary component was CaO, making up 88.75% of the total, along with minor amounts of SiO_2_, Al_2_O_3_, Fe_2_O_3_, and SO_3_. SS was sourced from a metallurgical plant in Shanxi Province. The main chemical components were Fe_2_O_3_, SiO_2_, and CaO, with additional amounts of Al_2_O_3_, SO_3_, K_2_O, and Na_2_O. PC was commercially available PI 42.5 ordinary Portland cement, primarily composed of CaO and SiO_2_. Additionally, ordinary tap water was used throughout the experiments to ensure smooth procedures and reliable results. The detailed chemical compositions of all materials are comprehensively listed in Table 1.

**Figure 1:**
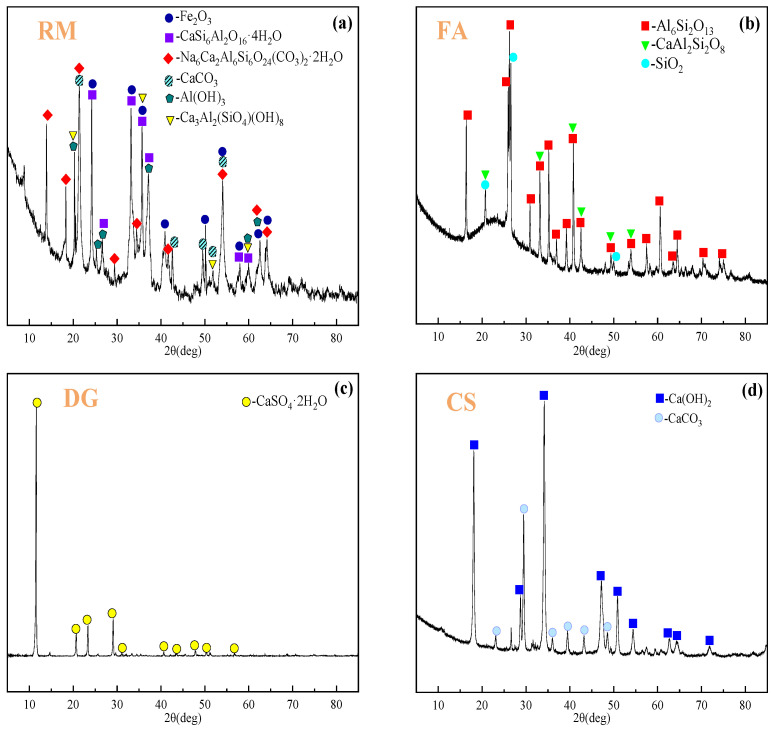

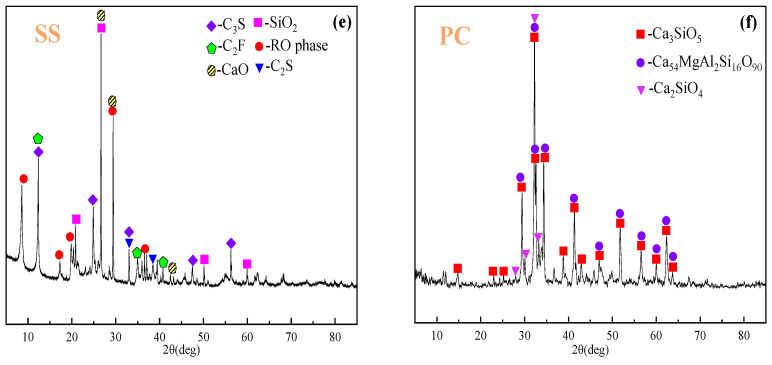
XRD patterns of RM (**a**), FA (**b**), DG (**c**), CS (**d**), SS (**e**), and PC (**f**).

In mineralogical terms, RM exhibits a complex and diverse composition, primarily comprising Fe_2_O_3_, CaSi_6_Al_2_O_16_·4H_2_O, CaCO_3_, Ca_3_Al_2_(SiO_4_)(OH)_8_, Al(OH)_3_, and Na_6_Ca_2_Al_6_Si_6_O_24_(CO_3_)_2_·2H_2_O. FA demonstrates a more concentrated mineralogical profile, dominated by SiO_2_, CaAl_2_Si_2_O_8_, and Al_6_Si_2_O_13_. The mineral phase of DG is mainly CaSO_4_·2H_2_O. CS predominantly consists of Ca(OH)_2_, accompanied by minor amounts of CaCO_3_. SS presents the most intricate mineralogical composition, encompassing C_2_S, C_3_S, C_2_F, RO phase (a complex solid solution containing MgO, FeO, MnO, and other components), and CaO. PC is primarily composed of C_2_S and C_3_S. The mineral phase compositions of RM, FA, DG, CS, SS, and PC were analyzed using X-ray diffraction (XRD), with their corresponding XRD patterns illustrated in Figure 1.

**Figure 2:**
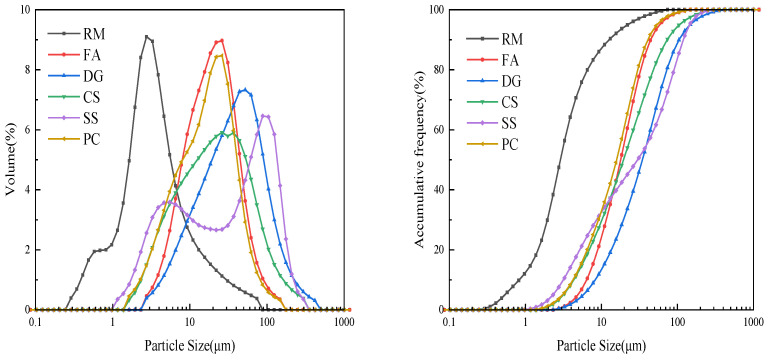
The particle size distribution curves of various raw materials.

The particle size distribution of each raw material was analyzed using laser diffraction with a Microtrac S3500 instrument (Microtrac, York, PA, USA), with the results presented in Figure 2. The average particle sizes were determined as follows: RM at 5.32 µm, with a median particle size (d_50_) of 2.87 µm; FA at 22.91 µm, with a d_50_ of 17.92 µm; DG at 49.63 µm, with a d_50_ of 34.68 µm; CS at 32.38 µm, with a d_50_ of 19.54 µm; SS at 48.44 µm, with a d_50_ of 30.00 µm; and PC at 20.40 µm, with a d_50_ of 15.87 µm.

### 2.2. Experimental Procedure

The experimental design established red mud, fly ash, and cement as fixed cementitious components. Previous laboratory studies have demonstrated that the formulated red mud composite backfill with 70% red mud content meets mine backfilling requirements, while excessive dosage would lead to compromised material strength [38]. Therefore, this experiment sets the red mud proportion at 70%. Accordingly, the proportions of RM, FA, and cement were set at 7:2:1. Desulfurized gypsum (DG), carbide slag (CS), and steel slag (SS) were employed as supplementary activators. A reference baseline system was established using an RM–FA–cement ratio of 7:2:1, and the effects of different industrial solid waste activators were investigated at incorporation levels of 4%, 6%, and 8%. Studies indicate that when the water-to-cement (w/c) ratio in mortar exceeds 0.5 [39], compressive strength decreases with increasing w/c. Therefore, considering both workability and strength, the w/c ratio was set at 0.5. Mortar specimens of the corresponding composite systems were prepared, including the RM–FA–cement system (RFC), RM–FA–DG–cement system (RFDC), RM–FA–CS–cement system (RFCC), and RM–FA–SS–cement system (RFSC), to investigate the synergistic effects and activation mechanisms of different solid wastes with RM.

The experimental procedure was conducted as follows: Initially, component materials including red mud, fly ash, cement, desulfurization gypsum, carbide residue, and steel slag were precisely weighed using an electronic balance (YP-6002; Tianjin Tianma Hengji Instrument Co., Ltd., Tianjin, China) according to pre-established mixing ratios. A handheld mixer was employed for dry blending (duration: 5 min) to produce the precursor of the red mud-based filling material. Subsequently, deionized water was added at a water-to-cement ratio of 0.5, followed by thorough re-mixing (duration: 10 min) to obtain the final red mud-based filling material. The cementitious samples were then placed in a curing chamber (YH-60B; Beijing CCCC Construction Instrument Technology Development Co., Ltd., Beijing, China) for standardized curing durations of 3, 7, and 28 days. After curing, the uniaxial compressive strength was tested using a 100 kN microcomputer-controlled electronic universal testing machine (DKDW-10D; Xuzhou Dongkong Technology Co., Ltd., Xuzhou, China).

**Figure 3:**
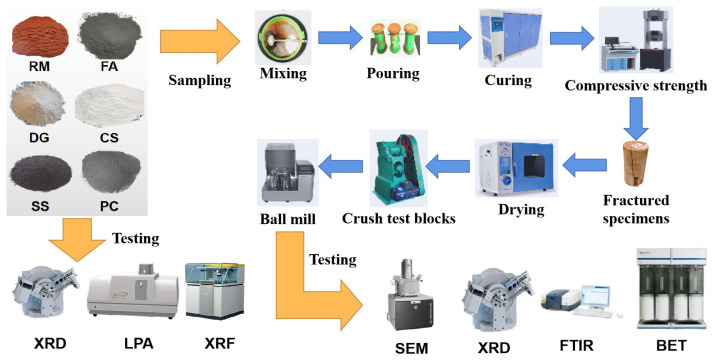
Experimental flowchart.

Based on the preliminary screening of the experimental results, specimens with superior performance were selected from each system, and core samples were extracted from these specimens after uniaxial compression. Subsequently, the sampled materials were finely crushed using a jaw crusher (EP-2; Hebi Innovative Instrumentation Co., Ltd., Hebi, China). The crushed materials were then placed in an electric digital display drying oven (101-2A; Hebi City Innovative Instrumentation Co., Ltd., Hebi, China) and dried at a constant temperature of 80 °C for 24 h. After drying, the samples were transferred to stainless steel jar containers of a planetary ball mill (YXQM-4L; Changsha Miqi Instrument and Equipment Co., Ltd., Changsha, China) equipped with stainless steel grinding balls. High-efficiency grinding was conducted at 500 revolutions per minute (rpm) for 10 min. Finally, the processed materials were sieved through a 200-mesh sieve to obtain refined powders for subsequent analyses, including XRD, SEM, BET, FTIR, environmental performance testing, and further research. A detailed experimental flowchart is presented in Figure 3, and the specific material formulations are summarized in Table 2.

### 2.3. Experimental Methods

#### 2.3.1. Uniaxial Compressive Strength Test

Samples were poured into standard cylindrical molds (50 mm × 100 mm) fixed on a vibration table for molding. Nine specimens were prepared for each group and demolded after 24 h. The specimens were then labeled and placed in a curing chamber (Model YH-60B) under controlled conditions (95% relative humidity, 22 °C) for curing periods of 3 d, 7 d, and 28 d. Uniaxial compression tests were conducted using a DKDW-10D microcomputer-controlled electronic universal testing machine (Xuzhou Dongkong Technology Co., Ltd., Xuzhou, China). The average strength of three specimens of each system specimen in different curing periods was recorded as the final result [38].

#### 2.3.2. Microstructure Characterization

The total pore structure of the powdered samples was analyzed using a Brunauer–Emmett–Teller (BET) surface area and porosity analyzer. Samples were pretreated under vacuum conditions using a Micromeritics degassing station (Micromeritics, Norcross, GA, USA). Nitrogen adsorption–desorption tests were then performed at 77 K with a Micromeritics APSP 2460 automatic specific surface and porosity analyzer. The resulting isothermal adsorption–desorption curves and pore size distribution profiles were obtained for further analysis [40]. The machine model used for BET analysis in this test was the Micromeritics ASAP 2460 automatic specific surface and porosity analyzer (Micromeritics, GA, USA).

The mineral phases of 28-day cured samples were characterized by X-ray diffraction (XRD) using a Cu Kα radiation source. Scans were conducted in the 2θ range of 5–90° at a rate of 2°/min. The hydration products were qualitatively analyzed using Jade Software v6.0 [38].

Appropriate amounts of dried sample and potassium bromide (KBr) powder were mixed in an agate mortar and ground thoroughly. The mixture was pressed into transparent pellets using a hydraulic press. Background scans were first collected, followed by sample scans at a resolution of 4 cm^−1^ with 32 accumulations over the wavenumber range of 400–4000 cm^−1^. FTIR spectra were recorded for functional group identification [41]. The model of the machine used for FTIR analysis in this test is the American Thermo Fisher Scientific Nicolet 20 Fourier-transform infrared spectrometer (Waltham, MA, USA).

The microstructures of ethanol-treated and vacuum-metallized samples were observed using a scanning electron microscope (SEM) at an accelerating voltage of 3 kV. The morphologies of the mixed and cured specimens were analyzed to evaluate their microstructural evolution [38]. The machine model used for SEM analysis in this test was a Zeiss Geminisem 300 scanning electron microscope (ZEISS, Oberkocken, Germany).

#### 2.3.3. Environmental Performance Test

The toxic leaching test of the test piece was carried out in accordance with the methods and steps of the national standard horizontal oscillation method for the toxic leaching of solid waste (HJ 557-2010 [42]). An NexION 1000 ICP-MS inductively coupled plasma atomic emission spectrometer (PerkinElmer, Waltham, MA, USA) was used to determine the content of harmful ions in the leaching solution, and the environmental performance of the test piece was analyzed [38].

## 3. Results and Discussion

### 3.1. Physicochemical Performance Analysis

#### 3.1.1. Compressive Strength Analysis

**Figure 4:**
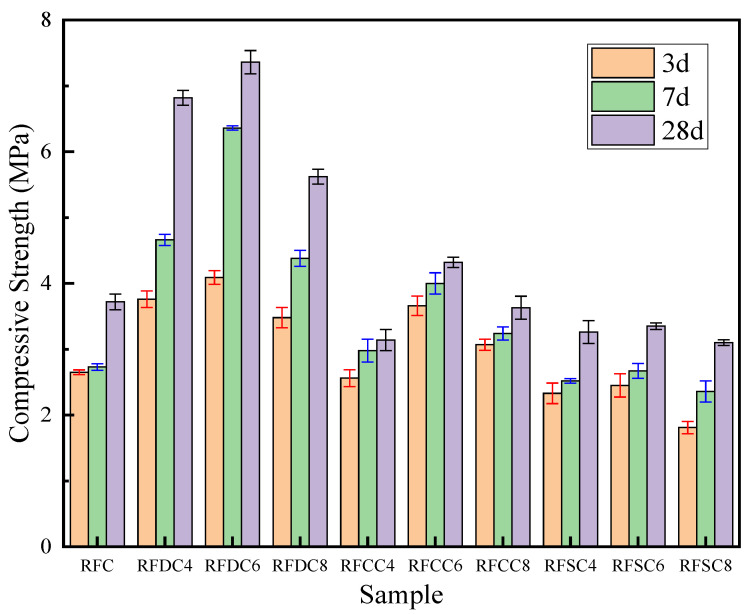
Compressive strength of each sample.

The compressive strength test results shown in Figure 4 demonstrate that the red mud-based backfill materials exhibit pronounced time-dependent behavior and activator concentration sensitivity. As the curing time extended from 3 days to 28 days, all specimens displayed a sustained increasing trend in compressive strength, indicating that hydration reactions persisted throughout the entire curing process. Notably, the compressive strength of the composite systems initially increased and then decreased with higher dosages of the three types of industrial solid waste activators. When the content of DG, CS, and SS is 6%, the effect is the best. See Table 3 for the change of compressive strength and increment of each sample.

The reference system RFC exhibited a 3-day compressive strength of 2.65 MPa, reflecting the inherent limitations of the red mud–fly ash–cement ternary system, where the release rate of pozzolanic active substances was slow. In contrast, the RFDC6 system with 6% added desulfurized gypsum demonstrated significant advantages, achieving a 3-day strength of 4.09 MPa, which represents a 54.3% increase compared to RFC. This early-stage enhancement is attributed to the chemical activation effect of sulfate ions (SO_4_^2−^) from the desulfurized gypsum: on one hand, SO_4_^2−^ rapidly reacts with Al^3+^ in red mud to form ettringite (AFt), creating an early strength framework; on the other hand, the Ca^2+^ released from gypsum dissolution accelerates the dissociation of glassy phases in the system through charge balance, promoting the dissolution of active SiO_2_ and Al_2_O_3_. The RFCC6 system with carbide slag achieved a 3-day strength of 3.66 MPa, representing a 38.1% improvement over the reference system. Its strengthening mechanism primarily relies on the alkaline environment provided by Ca(OH)_2_ which increases the system’s pH and partially activates the aluminate phases in red mud [43]. However, the RFSC6 system with steel slag showed a 3-day strength of 2.45 MPa, which is 7.5% lower than that of the reference system. This is attributed to the low reactivity of silicate minerals (C_2_S, C_3_S) in steel slag, which are unable to effectively participate in hydration reactions during the early stages [44].

During the 7-day curing period, the RFC system exhibited a compressive strength of 2.73 MPa, showing minimal improvement compared to its early-stage strength. In contrast, the RFDC6 system demonstrated continued significant strength development, with its compressive strength increasing to 6.36 MPa, representing a 133% enhancement over the RFC system at the same age. This progression can be attributed to the sustained release of sulfate ions (SO_4_^2−^), which reacted with dissolved Al_2_O_3_ in the composite system to form an ettringite network while simultaneously promoting the densification of C-S-H gel growth. The carbide slag-based RFCC6 system displayed slowed strength growth at this stage, achieving a compressive strength of 4.00 MPa, which corresponds to a 46.5% increase compared to RFC. This deceleration is attributed to the near-complete consumption of active components (Ca(OH)_2_) in the carbide slag and the inhibitory effects of impurities on silicate phase hydration. Notably, the steel slag-based RFSC6 system reached a compressive strength of 2.67 MPa, remaining 2.2% lower than that of RFC. However, an 8.9% strength increase compared to its 3-day performance suggests the initial participation of reactive minerals in steel slag (e.g., C_2_S, C_3_S) in hydration reactions.

During the 28-day curing period, the RFDC6 system exhibited further strength growth to 7.36 MPa, marking a 97.8% increase compared to the RFC system, demonstrating sustained hydration-driven reinforcement. At this stage, a multiscale reinforced structure formed within the material: micron-scale ettringite crystals synergized with nano-sized C-S-H gel to enhance matrix densification through pore-filling effects. In contrast, the RFCC6 system achieved a 28-day strength of only 4.32 MPa, representing a 16.1% improvement over the reference system but a significantly lower value than its mid-term growth rate. This indicates the time-limited effectiveness of carbide slag’s alkaline activation mechanism. While the initial pH elevation partially activated aluminate phases, the lack of continuous ion supply led to insufficient reaction kinetics in later stages. Notably, the RFSC6 system ultimately reached a strength of 3.35 MPa, remaining 9.9% lower than that of the reference system. This confirms that steel slag fails to effectively activate the pozzolanic reactivity of red mud, likely due to the sluggish dissolution kinetics of its silicate phases under the system’s chemical environment.

A further in-depth analysis of the difference in compressive strength between 3 days and 28 days showed that the 3-day strength of RFC was 2.65 MPa, and the 28-day strength increased to 3.72 MPa, with a growth rate of 40.4%. The 3-day strength of RFDC6 was 4.09 MPa, and the 28-day strength was 7.36 MPa, with a growth rate of 80.0%, which was significantly better than those of other systems. The 3-day strength of RFCC6 was 3.66 MPa, and the 28-day strength increased to 4.32 MPa, with a growth rate of only 18.0%. The 3-day strength of RFSC6 was 2.45 MPa, but the 28-day strength increased to 3.35 MPa, with a growth rate of 36.7%, which was slightly better than that of RFC. By comparing the strength growth rate of 3 days and 28 days, the time effect of different activators can be further analyzed. Although the growth rate of the RFC system reached 40.4%, its absolute increment was only 1.07 MPa, reflecting that the hydration reaction of the basic system was mainly concentrated in the early stage. The enhancement ability of the RFDC6 system runs through the whole process and continuously stimulates the pozzolanic activity of red mud from 3 d to 28 d. The growth rate of RFCC6 was only 18.0%, indicating that the effect of carbide slag on the activation of the red mud’s pozzolanic activity was limited. Although the growth rate of RFSC6 is 36.7%, its initial strength is too low, and its final performance is still lower than that of the benchmark system RFC.

#### 3.1.2. Energy Evolution Patterns

**Figure 5:**
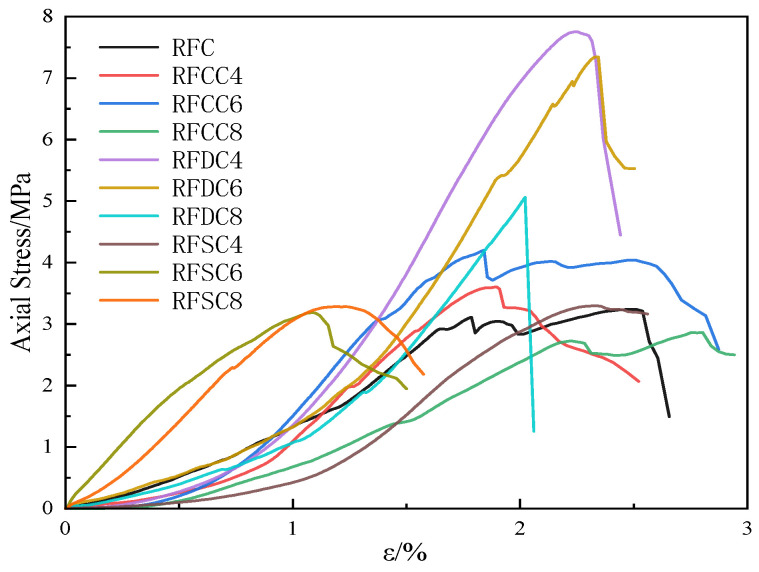
Stress–strain curve.

The characteristics of the stress–strain curves obtained from the tests are illustrated in Figure 5 below. As shown, all specimen groups exhibited essentially linear deformation characteristics prior to reaching peak strength. The specimens of the RFDC and RFSC systems displayed slight downward curvature near failure, followed by a sharp decline during the failure phase, indicating predominantly brittle behavior. In contrast, the RFC and RFDC systems demonstrated noticeable post-peak yielding, with significant strain development accompanied by marginal stress variation, characterizing their elastoplastic properties [45].

According to the first law of thermodynamics, the input energy of a specimen under uniaxial compression is primarily converted into elastic energy and dissipated energy [46]. The energy indicators of the specimen are calculated as follows:

(1) U=∫σdε

(2) $$U_{e}=\sigma^{2}/2E$$

(3) $$U_{d}=U-U_{e}=\int \sigma d\varepsilon -\sigma^{2}/2E$$

where *U*, *U_e_*, and *U_d_* are input energy density, elastic energy density, and dissipation energy density, respectively, J/cm^−3^; *E* is the tangent elastic modulus, MPa; σ is the axial stress, MPa; and ε is the axial strain, MPa.

**Figure 6:**
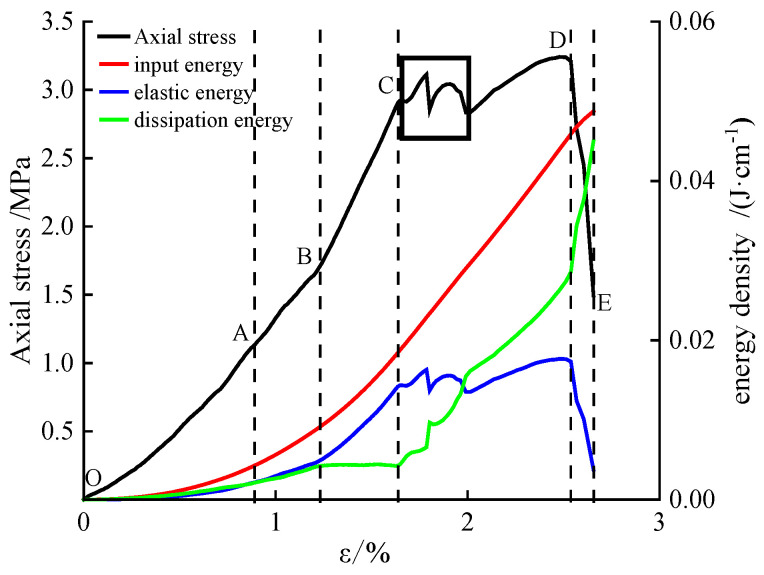
Stress–strain and energy curves of 28-day RFC sample.

Since the energy evolution patterns during the damage and failure processes of all specimens are similar, and due to space limitations, the energy evolution characteristics of the 28-day RFC specimen are utilized as an example to analyze the energy evolution patterns during specimen damage and failure. Based on existing research, the energy curve can be roughly divided into five stages [46,47], as shown in Figure 6.

(1) Initial damage stage (OA). The dissipated energy increases nonlinearly with deformation, primarily due to the closure of micropores and internal friction within the specimen. Both the input energy and elastic energy also increase with deformation, but the elastic energy remains lower than the dissipated energy during this stage. As the micropores close, the growth rate of the elastic strain energy begins to accelerate. The specimen transitions into the stable damage stage when the elastic strain energy equals the dissipated energy (after the intersection point of the curves). Therefore, the point where the dissipated energy equals the elastic energy can be regarded as the boundary between the initial damage stage and the stable damage stage.
(2) Stable damage stage (AB). After the intersection point of the dissipated energy and elastic strain energy curves, the specimen enters the stable damage stage. During this stage, as the micropores are further compacted and internal friction continues, the dissipated energy curve increases approximately linearly. Both the total energy and elastic strain energy increase at the same rate, with their growth rates exceeding that of the dissipated energy. This stage is primarily characterized by the accumulation of elastic strain energy.
(3) Stationary damage stage (BC). As the micropores become fully compacted, the specimen enters the stationary damage stage. During this stage, the dissipated energy remains nearly constant, and its evolution curve appears approximately horizontal. At this point, the micropores in the rock sample are completely closed, and almost no new dissipated energy is generated. As a result, the slope of the dissipated energy evolution curve is minimal (close to zero). Meanwhile, both the total energy and elastic energy continue to increase at an accelerated rate as the rock sample is further compacted, with their growth trends remaining consistent. In this stage, the energy input into the rock sample is primarily converted into elastic energy.
(4) Accelerated damage stage (CD). As the deformation of the fractured rock sample increases, internal cracks begin to propagate and new cracks form, leading to the dissipation of the elastic strain energy stored in the rock sample. During this stage, the growth rate of the dissipated energy gradually increases (indicated by the rising slope of the dissipated energy evolution curve), while the growth rate of the elastic strain energy gradually decreases (indicated by the declining slope of the elastic strain energy evolution curve). As the specimen reaches its maximum compressive strength, the total input energy essentially peaks, and the elastic strain energy stored in the rock sample reaches its limit. At this point, the damage evolution of the fractured rock mass enters the failure stage.
(5) Damage failure stage (DE). After the rock sample reaches its peak strength, the damage evolution of the specimen enters the damage failure stage. During this stage, the dissipated energy evolution curve exhibits a sharp and sudden increase, while the elastic strain energy evolution curve shows a sharp and sudden decrease, and the total energy remains essentially constant. The further propagation and interconnection of cracks in the fractured rock mass cause the specimen to ultimately lose its load-bearing capacity. The elastic strain energy accumulated in the rock sample is rapidly released in the form of dissipated energy, resulting in a sharp increase in the dissipated energy curve and a sharp decrease in the elastic strain energy curve [48].

After the specimen enters the accelerated damage stage, the elastic energy curve exhibits a distinct “double-peak” characteristic [49]. Upon reaching the initial peak, the elastic strain energy suddenly decreases in a step-like manner, while the dissipated energy suddenly increases in a step-like manner. During the deformation process, the presence of cracks leads to stress concentration in these regions, causing cracks to initiate and propagate. The elastic strain energy accumulated in the specimen is suddenly released, resulting in a sharp decrease in elastic strain energy and a sharp increase in dissipated energy. As the cracks propagate upward and downward, they cause two stress drops, significantly damaging the specimen. The load, initially borne by the entire rock sample, is transferred to the supporting blocks formed after crack propagation, leading to a substantial reduction in load-bearing capacity. Consequently, the elastic strain energy continues to increase with strain, but its growth rate begins to decrease. Eventually, due to friction between fracture surfaces and the formation of new macroscopic cracks, the elastic strain energy accumulated in the rock sample is continuously released, and the growth rate of dissipated energy rapidly increases, causing the specimen to ultimately lose its load-bearing capacity. This is why the specimen exhibits a “double-peak” shape at the peak point.

**Figure 7:**
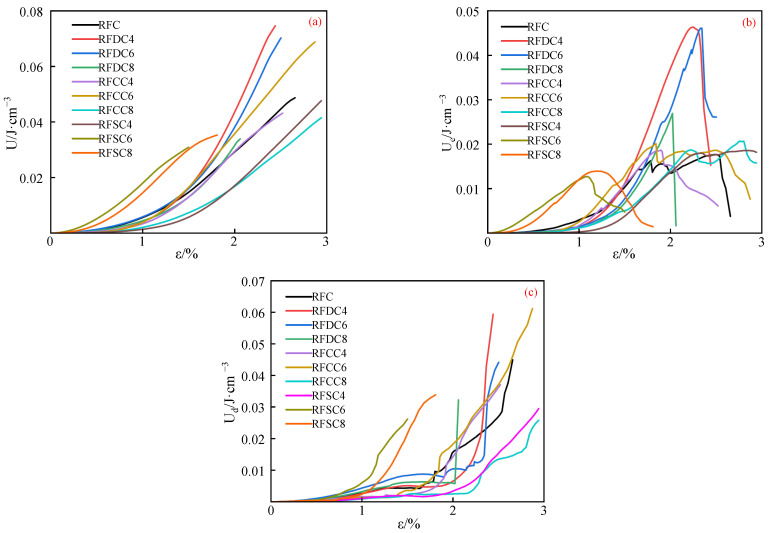
Strain–energy curves of various 28-day cured specimens ((**a**): input energy; (**b**): elastic energy; (**c**): dissipation energy).

The input energy, elastic energy, and dissipated energy of each group of specimens are shown in Figure 7. It can be observed that the input energy, elastic energy, and dissipated energy of the RFSC system specimens are generally lower than those of the other systems, indicating that the mechanical properties of the RFSC system specimens, such as elasticity, toughness, and compressive strength, are inferior to those of the other systems. This reflects their insufficient energy absorption and load-bearing capacity, as well as their lower overall structural integrity. In contrast, the input energy, elastic energy, and dissipated energy of the RFDC and RFCC system specimens are generally superior to those of the RFC reference system. This demonstrates that the addition of desulfurized gypsum or carbide slag to red mud-based backfill materials can enhance their performance, enabling them to effectively absorb and dissipate energy under external forces, thereby reducing deformation or damage [48].

As shown in Figure 7b, in the RFDC system specimens, the elastic energy exhibits a sharp decline after reaching its peak. This phenomenon indicates that the RFDC system specimens are relatively intact and possess a higher elastic energy release rate, leading to immediate and severe failure upon reaching the ultimate state. In contrast, the elastic energy of specimens from other systems decreases gradually, suggesting the presence of numerous internal cracks. During the process of crack destruction and subsequent compaction, a “double-peak” phenomenon occurs. Macroscopically, this manifests as the specimens’ ability to continue absorbing energy after reaching the ultimate state, with a slow release of elastic energy.

From Figure 7c, it can be seen that the dissipated energy curve shows a nonlinear increasing trend (concave shape) before the inflection point as deformation increases [50]. This is primarily caused by the closure of microcracks within the specimen and friction. As the deformation of the specimen increases, the cracks at the tips of the fissures further propagate and generate new cracks, leading to the dissipation of elastic strain energy stored in the specimen. The rate of growth of dissipated energy gradually increases (the slope of the dissipated energy evolution curve gradually increases), while the rate of growth of elastic strain energy gradually decreases (the slope of the elastic strain energy evolution curve gradually decreases). When the slope of the dissipated energy curve increases to positive infinity and the slope of elastic strain energy decreases to zero, the total input energy essentially reaches its peak value, and the elastic strain energy stored in the rock sample reaches its limit. The damage evolution of the fissured rock mass enters the damage failure stage. Existing research indicates that the energy dissipation effect of the specimen can be evaluated by the difference between the strain value corresponding to the maximum dissipated energy in the dissipated energy curve and the strain value corresponding to the inflection point of dissipated energy, which is defined as the dissipated energy step length. It can be observed that the dissipated energy step length of RFDC6 is the shortest, indicating that it has fewer internal microcracks and does not dissipate a large amount of energy before fracturing. Until it reaches the limit state, the elastic energy can be rapidly released and converted into dissipated energy. In contrast, the systems with a “dual-peak” phenomenon, such as RFC and RFCC, exhibit significantly longer dissipated energy step lengths, which also indicates that they have better elasticity and can maintain the integrity of the filling body structure, preventing structural collapse due to local overload.

### 3.2. Microstructure Analysis

Based on the results of the uniaxial compressive strength tests and energy evolution analysis, materials with a 6% mass fraction of industrial solid waste activators were selected for in-depth investigation and comparison with the reference system. Through a microstructural analysis of these materials, the synergistic effects and activation mechanisms between DG, CS, SS, and RM were revealed.

#### 3.2.1. Analysis of Hydration Product Phases

**Figure 8:**
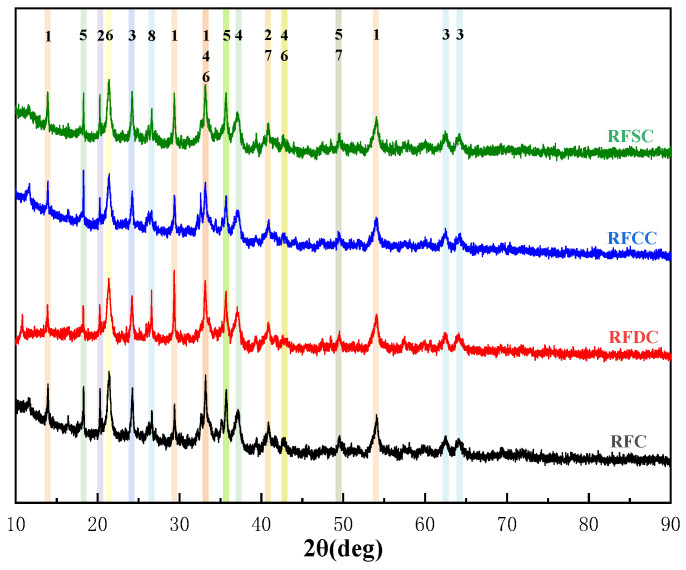
XRD patterns of RFC, RFDC, RFCC, and RFSC after 28 days of curing. Notes: 1. C(-A)-S-H gel. 2. Quartzite. 3. Hematite. 4. Ettringite. 5. Phillipsite. 6. Calcium hydroxide. 7. Calcite. 8. Scawtite.

The mineral compositions of the selected four groups of red mud-based backfill materials were analyzed using XRD technology. The XRD patterns of the cementitious materials from each group after 28 days of standard curing are shown in Figure 8. The results indicate that the mineral phase compositions of all groups are highly consistent overall. The main hydration products include C-S-H gel (CaO·xSiO_2_·yH_2_O), quartz (SiO_2_), hematite (Fe_2_O_3_), ettringite (3CaO·Al_2_O_3_·3CaSO_4_·32H_2_O), gismondine (CaAl_2_Si_2_O_8_·4H_2_O), calcium hydroxide (Ca(OH)_2_), calcite (CaCO_3_), and lawsonite (CaAl_2_[Si_2_O_7_](OH)_2_·H_2_O). This suggests that desulfurized gypsum, carbide slag, and steel slag do not directly react with red mud but instead act as catalysts to activate the pozzolanic reactivity of red mud. Additionally, it is noteworthy that hematite, as a direct product of bauxite dissolution under strong alkaline conditions, is chemically stable and does not participate in the hydration reactions of the material.

During the early hydration process of the specimen, C_2_S and C_3_S in the cement rapidly dissolve and react with water to produce C-S-H gel and Ca(OH)_2_. Subsequently, the reactive silicoaluminate materials in red mud and fly ash, under the influence of an alkaline environment, lead to the destruction of the covalent bonds Si-O-Si and Al-O-Al in their mineral glass phases. This results in the formation of free reactive species Si^4+^ and Al^3+^ ions, which further generate [H_3_SiO_4_]^−^ and [H_3_AlO_4_]^2−^ ions. As the concentrations of [H_3_SiO_4_]^−^ and [H_3_AlO_4_]^2−^ ions in the reaction system gradually increase, a certain concentration of [H_3_SiO_4_]^−^ and [H_3_AlO_4_]^2−^ ions will combine with Ca^2+^ to form C-A-S-H gel. The involved chemical reaction equations can be expressed as follows [51]:

C_3_S + H_2_O → C-S-H + Ca(OH)_2_ (4)

C_2_S + H_2_O → C-S-H + Ca(OH)_2_ (5)

SiO_2_ + OH^−^ + H_2_O → [H_3_SiO_4_]^−^ (6)

Al_2_O_3_ + OH^−^ + H_2_O → [H_3_AlO_4_]^2−^ (7)

[H_3_SiO_4_]^−^ + [H_3_AlO_4_]^2−^ + Ca^2+^ → C-A-S-H (8)

A further comparison of the four groups of materials reveals that the peak intensity at approximately 30° in the RFDC6 specimen is higher than that of the other specimens, indicating that it generates more C(-A)-S-H gel. This is because, after the addition of desulfurization gypsum, the reactive silicoaluminate materials in the raw materials are dissolved into the liquid phase of the sample under the synergistic action of OH^−^ and SO_4_^2−^ ions. These materials are primarily composed of [SiO_4_] and [AlO_4_] tetrahedra, which combine with free calcium ions (Ca^2+^), sulfate ions (SO_4_^2−^), and sodium ions (Na^+^) in the system to form ettringite, amorphous gel (C(N)-A-S-H), and other silicate aluminates [52]. The elevated Ca^2+^ and SO_4_^2−^ content effectively promoted the formation of ettringite and amorphous gel (C(N)-A-S-H), consistent with the research findings of Vello Pallav [53]. These gel substances and crystals significantly enhance the overall connectivity and structural strength of the material. Additionally, ettringite can serve as a skeletal structure for the material, further improving the compressive strength of the cementitious material. The chemical reaction equations involved in this process can be expressed as follows [54]:

AlO_2_^−^ + OH^−^ + H_2_O → [Al(OH)_6_]^3−^ (9)

6Ca^2+^ + 3SO_4_^2−^ + 2[Al(OH)_6_]^3−^ + 26H_2_O = Ca_6_Al_2_(SO_4_)_3_(OH)_12_·26H_2_O (10)

[H_3_SiO_4_]^−^ + [H_3_AlO_4_]^2−^ + Ca^2+^ + Na^+^ → C(N)-A-S-H (11)

C-S-H gel and ettringite, as critical contributors to early strength, play a vital role in hydration reactions. Their formation often mutually promotes each other. Specifically, when red mud and cement dissolve in water, they create an alkaline environment that effectively dissolves reactive silicon and aluminum compounds, forming free reactive species. This process activates the pozzolanic reactivity of red mud and fly ash. Simultaneously, Ca(OH)_2_ generated from cement hydration not only enhances the alkaline environment during the reaction but also [55], under the synergistic effect of SO_4_^2−^ ions, participates as a reactant in subsequent hydration processes, further promoting the formation of ettringite. As C(-A)-S-H gel and ettringite are generated, the C(-A)-S-H gel adheres to the ettringite, interweaving to form a network structure. This network continuously fills the interstitial pores as the reaction progresses. This not only enhances the structural stability of the material, creating a dense matrix, but also allows free ions to attach to the dense structure, enabling secondary hydration reactions to proceed fully.

#### 3.2.2. Chemical Bond Analysis of Hydration Products

**Figure 9:**
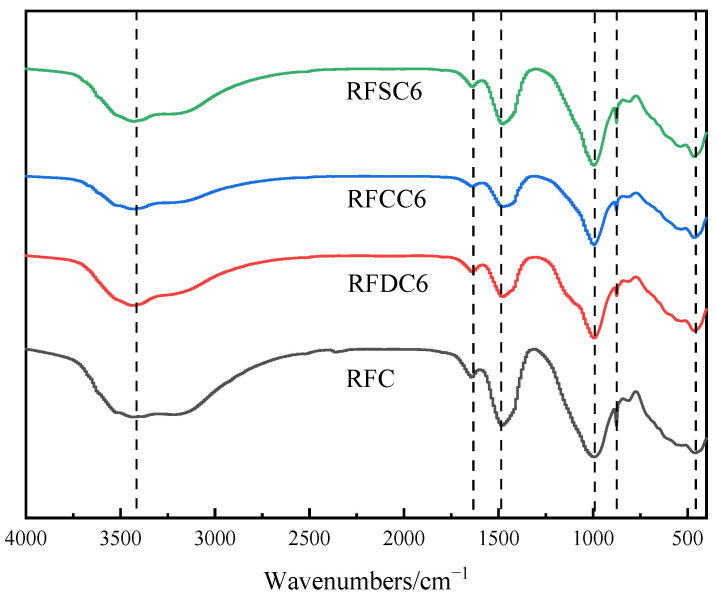
FTIR spectra of various specimens after 28-day hydration.

Fourier-transform infrared spectroscopy (FTIR) was used to detect and analyze the chemical structural changes in each group of specimens. The FTIR spectra of the hydration products of the samples at 28 days are shown in Figure 9. From the figure, the absorption peak at 3432 cm^−1^ is attributed to the stretching vibration of O-H. The absorption peaks at 1638 cm^−1^ and 1477 cm^−1^ correspond to the stretching vibration and asymmetric stretching vibration of C-O, respectively. The absorption peak at 995 cm^−1^ corresponds to the stretching vibration of Si-O-Si. In the range of 600 cm^−1^ to 800 cm^−1^, the absorption peaks correspond to the symmetric stretching vibrations of Si-O-Si or Si-O-Al in [SiO_4_]^4−^ or [AlO_4_]^5−^ tetrahedra. The peak at 875 cm^−1^ corresponds to the Ca-O vibration, and the absorption peak at 462 cm^−1^ corresponds to the bending vibration of Si-O-Si or Si-O-Al. It can be observed that the RFC specimen exhibits slightly higher peaks for the Si-O-Si stretching vibration at 995 cm^−1^ and the asymmetric stretching vibration of C-O at 1477 cm^−1^ compared to the other samples.

Based on the characteristics of Si-O stretching vibrations in silicate materials, the characteristic absorption peak positions in the infrared spectrum corresponding to SiQ^n^ structural units (where Q represents tetrahedral Si, and n is the number of bridging oxygens) with different degrees of polymerization are as follows: SiQ^0^ (n = 0, ~850 cm^−1^), SiQ^1^ (n = 1, ~900 cm^−1^), SiQ^2^ (n = 2, ~950 cm^−1^), SiQ^3^ (n = 3, ~1100 cm^−1^), and SiQ^4^ (n = 4, ~1200 cm^−1^) [56]. As seen in the figure, the characteristic peak appearing at 995 cm^−1^ in the red mud-based filling material indicates the coexistence of SiQ^2^ and SiQ^3^ structures within the system. This phenomenon suggests that the calcium silicate hydrate (C-S-H) gel formed from the red mud-based filling material has structural characteristics similar to those of the aluminosilicate gel formed in alkaline-activated systems. It can be inferred that the hydration products of the red mud-based filling material have formed a C-A-S-H gel with a higher degree of polymerization, where the silicate tetrahedral structure primarily exists in the form of SiQ^2^ and SiQ^3^ units.

#### 3.2.3. Pore Structure Analysis

**Figure 10:**
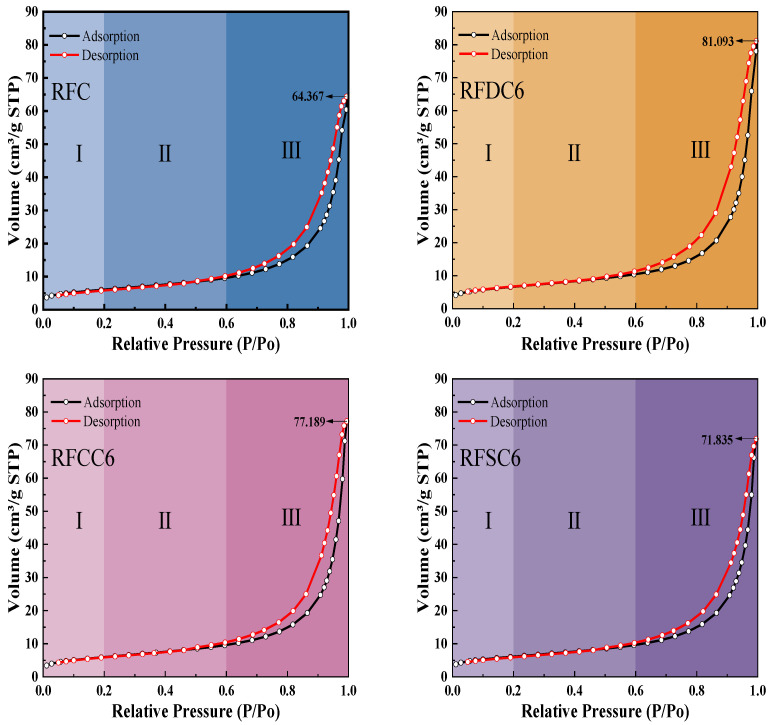
Adsorption–desorption isotherms of specimens after 28-day curing.

Pore structure is one of the critical components of cementitious materials. In this experiment, a fully automated specific surface area and porosity analyzer (BET) was used to conduct nitrogen adsorption–desorption tests on four selected sample groups under liquid nitrogen conditions at 77 K. The analysis produced isothermal adsorption–desorption curves, as illustrated in Figure 10. According to the Brunauer–Deming–Teller classification, the obtained isothermal adsorption–desorption curves are classified as Type II, indicating the presence of multilayer adsorption phenomena of N_2_. These isothermal adsorption–desorption curves are divided into three stages: Stage I (P/P_0_ < 0.2), Stage II (0.2 < P/P_0_ < 0.6), and Stage III (0.6 < P/P_0_ < 1).

In the first stage (Stage I), N_2_ forms typical monolayer adsorption on the surface micropores of the red mud-based filling material. As the relative pressure increases, the system quickly enters the second stage (stable rising stage, Stage II), followed by a sharp rise in adsorption capacity (Stage III). The curvature changes at point B in the four sets of adsorption–desorption isotherms are not significant, indicating a notable overlap between the monolayer adsorption and the initial stage of multilayer adsorption. The sharp increase in the curvature of the adsorption–desorption isotherms is due to the gradual formation of a multilayer adsorption structure, which leads to unrestricted adsorption as the N_2_ saturation vapor pressure is approached. When P/P_0_ = 1, the thickness of the adsorbed multilayer film typically exhibits an unrestricted growth trend. At this vapor pressure, the N_2_ adsorption capacities of RFC, RFDC6, RFCC6, and RFSC6 reach 64.367 cm^3^/g, 81.093 cm^3^/g, 77.189 cm^3^/g, and 71.835 cm^3^/g, respectively. From the adsorption–desorption curves, the presence of an H3-type hysteresis loop (IUPAC classification) indicates the existence of slit-shaped and conical pore structures in the material [57].

**Figure 11:**
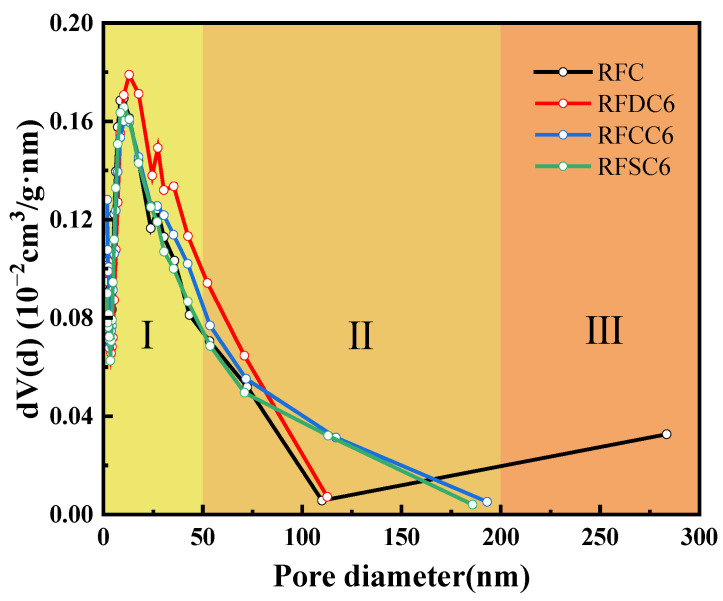
Pore size distribution curves of specimens after 28 days of curing.

The pore size distribution curves are shown in Figure 11. Based on the pore distribution and pore size of the four groups of specimens, they can be divided into three regions: Region I (2 nm < pore size distribution < 50 nm), Region II (50 nm < pore size distribution < 200 nm), and Region III (pore size distribution > 200 nm) [56]. The pores in Region I mainly include gel pores and small capillary pores, which primarily affect the strength, permeability, shrinkage, and creep properties of the cementitious material. According to the pore size distribution curves, the pores of the four groups of specimens are mainly distributed in Region I. The proportional area of Region I for the four specimens follows the order RFDC6 > RFCC6 > RFSC6 > RFC. The higher the proportion of gel pores and (small) capillary pores in the specimens, the denser the matrix, which is consistent with the trend in the compressive strength results. For the RFSC6 specimen, the presence of a significant amount of unreacted raw materials may result in some pores being intrinsic to the raw materials rather than gel pores or small capillary pores. Therefore, the densification degree of the RFSC6 specimen is lower than that of the RFC specimen, which aligns with the compressive strength and SEM analysis results. In Region II, the pores are mainly large capillary pores. The slopes of the pore size distribution curves for RFDC6 and RFC are steeper than those for RFCC6 and RFSC6, indicating that the number of large capillary pores in RFCC6 and RFSC6 is greater than in RFDC6 and RFC, thereby affecting the compressive strength of RFCC6 and RFSC6. In Region III, large pores are only present in the RFC specimen, making it more prone to damage and resulting in a lower compressive strength compared to RFCC6.

The specific surface area, total pore volume, and average pore size of each specimen are shown in Table 4. It can be observed that the order of specific surface area is RFDC6 > RFSC6 > RFC > RFCC6; the order of total pore volume is RFDC6 > RFCC6 > RFSC6 > RFC; and the order of average pore size is RFCC6 > RFSC6 > RFDC6 > RFC. Among them, RFDC6 has the largest specific surface area and total pore volume, but its average pore size is relatively small. This is because the RFDC6 specimen contains a significant number of gel pores and (small) capillary pores [58,59]. The abundance of these pores indicates a higher degree of cementation in the RFDC6 specimen compared to the others, resulting in its highest compressive strength [60]. The RFC specimen has the smallest total pore volume and average pore size, but its specific surface area is comparable to that of RFCC6 and RFSC6. This is due to the extreme distribution of pore sizes in the RFC specimen. Although it has fewer pores and generally smaller pore sizes, the presence of large pores ranging from 200 to 300 nm makes it more susceptible to damage.

#### 3.2.4. Micro Morphology Analysis

**Figure 12:**
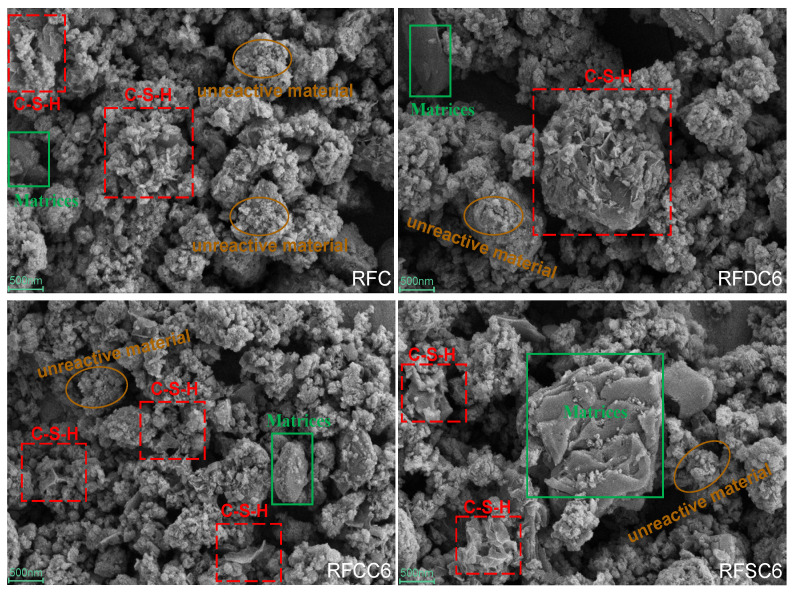
SEM images of each sample at 28 days.

To further investigate the microstructure of the hydration products in the cementitious materials, as well as the synergistic effects and activation mechanisms among desulfurized gypsum, carbide slag, steel slag, and red mud, scanning electron microscopy (SEM) analysis was conducted on samples cured for 28 days. The specific results are shown in Figure 12. The SEM images clearly illustrate the morphology of the hydration products in the four groups of samples. In all samples, the presence of C-S-H gel was observed, which is a significant contributor to the strength of the cementitious materials. Additionally, granular substances were found scattered on the surface of the final hydration products, which are likely residues from the raw materials that did not participate in the reaction.

By comparing the microstructures of the four groups of samples, it can be observed that the hydration products of the RFDC6 system exhibit a denser microstructure. This is primarily attributed to the addition of desulfurized gypsum, which promotes the formation of ettringite and more C(-A)-S-H gel. These gel substances tightly encapsulate the ettringite, filling the pores within the matrix of the cementitious material and significantly reducing the number of pores. As a result, the material’s density and compressive strength are enhanced. This finding aligns with the superior performance of the RFDC6 system specimens in compressive strength tests.

The microstructure of the RFCC6 system is similar to that of the RFC system. Combined with the changes in compressive strength, this indicates that carbide slag primarily accelerates the rate of the hydration reaction rather than significantly promoting the formation of hydration products. Therefore, although carbide slag is less effective than desulfurized gypsum in enhancing the strength and density of the material, its addition to red mud-based filling materials can effectively improve their early strength.

The microstructure of the RFSC6 system exhibits certain unique characteristics, with more unreacted raw materials remaining compared to the other systems. This indicates that the hydration reaction of the red mud-based filling material in the RFSC6 system is hindered. Although steel slag contains C_2_S and C_3_S components, their low content and encapsulation by other impurities in the steel slag make it difficult for them to react fully. This explains the presence of a significant amount of unreacted raw materials observed in the microstructural images. Consequently, even though C(-A)-S-H gel is formed in the RFSC6 specimen under alkaline conditions, the lack of a dense binding structure makes it more susceptible to external forces, leading to a decrease in compressive strength. This finding is consistent with the earlier analysis of compressive strength and energy evolution patterns.

### 3.3. Economic and Environmental Impacts

As a region abundant in industrial solid wastes, Shanxi Province demonstrates significant dual economic and utilization value in materials such as RM, FA, DG, CS, and SS. The economic advantages of these materials lie in near-zero raw material costs (with negative costs arising from disposal fees paid by some enterprises), with primary expenses concentrated in short-distance transportation, showing clear superiority over traditional raw materials. Among these, fly ash and desulfurization gypsum have achieved localized large-scale application due to mature utilization technologies. Red mud can be converted into cementitious materials through alkaline activation, while carbide slag serves as a low-cost calcium source, and steel slag partially substitutes for cement. Notably, under the “coordinated solid waste utilization” model, multi-material formulations reduce single-category waste treatment costs by 20–30% while enhancing mechanical properties, establishing a “waste–treat–waste” circular economy paradigm.

Meanwhile, RM, FA, DG, CS, and SS are industrial solid wastes containing elements harmful to the environment. Therefore, it is necessary to evaluate the environmental performance of red mud-based filling materials. Table 5 presents the test results of heavy metal ion concentrations in the leachate of the four groups of samples: RFC, RFDC6, RFCC6, and RFSC6. The leaching concentrations of heavy metal ions in the leachate after the hydration reaction of each group of samples comply with the limits specified in the GB 8978-1996 [61]. As shown in Table 4, the leaching concentrations of Cr, Pb, and Cu are significantly reduced, with the Pb leaching concentrations in RFDC6 and RFCC6 being below the detection limit. The experimental results demonstrate that the synergistic effects between red mud and various industrial solid wastes can effectively immobilize heavy metal ions in the material, indicating that red mud-based filling materials exhibit excellent environmental performance [38].

### 3.4. Mechanism Analysis

In the RFC system, the primary components of cement, tricalcium silicate (C_3_S), and dicalcium silicate (C_2_S) undergo hydration reactions. According to reactions (4) and (5), they transform into C-S-H (calcium silicate hydrate) gel and calcium hydroxide (Ca(OH)_2_). Simultaneously, the alkaline environment in the system promotes the breaking of Si-O-Si and Al-O-Al bonds in red mud and fly ash, activating their pozzolanic activity. This releases free Si^4+^ and Al^3+^ ions, leading to the formation of C-S-H (calcium silicate hydrate) gel. As the reaction progresses, the concentrations of [H_3_SiO_4_]^−^ (silicate ions) and [H_3_AlO_4_]^2−^ (aluminate ions) in the solution gradually increase. When the concentrations of these ions reach a certain level, they combine with Ca^2+^ and Na^+^ ions in the solution. According to reactions (6) to (11), secondary hydration reactions further produce C(N)-A-S-H gel and ettringite.

In the RFDC system, the addition of desulfurized gypsum introduces a significant amount of SO_4_^2−^ ions. Under the synergistic effect of SO₄^2−^ and OH^−^ ions, the active aluminum substances in the raw materials are further activated, converting more aluminate ions. These ions then react with Ca^2+^ and SO_4_^2−^ ions according to reactions (9) and (10), generating more ettringite. Ettringite not only increases the solid content of the system as a hydration product but also serves as a framework for the attachment of C(-A)-S-H gel. Together with C(-A)-S-H gel, it fills the voids in the system, thereby enhancing the overall connectivity and mechanical properties of the red mud-based filling material.

In the RFCC system, the incorporation of carbide slag elevates system alkalinity, accelerating the progression of reactions (6)–(8) and thereby activating the pozzolanic reactivity of red mud and fly ash. Under alkaline conditions, the formation rate of C(-A)-S-H gel is enhanced, resulting in improved early-stage strength of the backfill. However, the inherent alkalinity of red mud limits the overall alkaline environment modification via limited carbide slag addition. Furthermore, the high concentration of inert Fe_2_O_3_ components in RM inhibits monomer reconstruction reactions, significantly constraining the enhancement effectiveness [25].

In the RFSC system, however, distinct mechanisms are observed. Although the C_3_S and C_2_S components in steel slag undergo hydration reactions to form C-S-H gel during the initial reaction stage, their limited content and interference from impurities impede complete reaction, failing to sufficiently enhance compressive strength. Concurrently, the carbon sequestration capability of steel slag induces RO-phase minerals to react with atmospheric CO_2_ under alkaline conditions, generating CO_3_^2−^ ions that further react with Ca^2+^ to precipitate CaCO_3_. This process dualistically inhibits ettringite formation while producing sparse, low-strength CaCO_3_ precipitates. These subcritical precipitates cannot provide adequate nucleation sites for C(-A)-S-H gel growth, compromising the integration of solid-gel matrices and reducing material densification, collectively resulting in systematically lower compressive strength.

In summary, desulfurized gypsum, carbide slag, and steel slag have different effects on the hydration process and the performance of red mud-based filling materials. The addition of desulfurized gypsum significantly enhances the overall performance of red mud-based filling materials. Carbide slag effectively promotes the hydration reaction of red mud, improving the early strength of the filling materials. However, with the addition of steel slag, due to its low reactivity, it is difficult for steel slag to participate in the hydration reaction. Additionally, the carbon capture capability of steel slag leads to its reaction with CO_2_ from the air, which inhibits the hydration reaction of red mud-based filling materials, thereby reducing their overall performance.

## 4. Conclusions

In this study, the macroscopic mechanical properties of red mud-based filling materials were systematically analyzed through uniaxial compressive strength tests and energy evolution analysis. Additionally, XRD, FTIR, SEM, and BET tests were employed to reveal the microscopic mechanisms during the hydration process of red mud-based filling materials. The conclusions are as follows:

(1) RFDC6 exhibits excellent mechanical properties at different curing ages. The compressive strength of the material increases by 80.0% from 3 days to 28 days. Particularly at the 28-day curing age, due to the high integrity of the specimen and fewer microcracks, it demonstrates higher rigidity and can more effectively absorb and dissipate energy during the damage process. Its compressive strength is significantly higher than that of other systems. RFCC6 performs well in early strength improvement, but the compressive strength of the material only increases by 18.0% from 3 days to 28 days. At the 28-day curing age, its compressive strength improvement is limited, primarily enhancing the elastic properties of the material, which helps maintain the integrity of the filling structure and prevent structural collapse due to local overload. RFSC6 shows lower mechanical properties than the RFC reference system at different curing ages. Although the compressive strength of the material increases by 36.7% from 3 days to 28 days, at the 28-day curing age, its input energy, elastic energy, and dissipated energy are generally low. The insufficient energy absorption and load-bearing capacity, along with the lower overall structural integrity, result in less ideal mechanical performance.
(2) Through microscopic analysis, it can be observed that the main hydration products of red mud-based filling materials include C(-A)-S-H gel and ettringite. These products interweave and adsorb to form a dense structural network, which is the primary factor contributing to the improvement in the material’s mechanical properties. In the RFDC6 system, the synergistic effect of SO_4_^2−^ promotes the generation of more C(-A)-S-H gel and ettringite, filling the internal pore structure of the material and enhancing its density. This transforms larger pores into smaller gel pores, making the pore structure more complete and more conducive to improving compressive strength. In the RFCC6 system, the increase in OH^−^ concentration promotes the early formation of C(-A)-S-H gel. This gel fills the pores, converting larger pores into larger capillary pores. However, due to the limited solid phase content, the material’s density does not significantly improve, but the substantial gel content significantly enhances the material’s elastic properties. In the RFSC6 system, there is a considerable amount of unreacted raw materials. Carbonate precipitates are formed due to the carbon capture capability of steel slag, and there are gaps between the unreacted raw materials and the hydration products. The internal structure of the material is relatively fragmented, lacking a complete load-bearing structure, which results in a decline in various properties.
(3) By testing the leachate of materials from each system, it can be observed that the heavy metal ion concentrations in the leachate of red mud-based filling materials all comply with the limits specified in the “GB 8978-1996 Integrated Wastewater Discharge Standard”. The synergistic effects of desulfurized gypsum, carbide slag, and steel slag with red mud effectively immobilize heavy metal ions in red mud-based filling materials, demonstrating excellent environmental performance.

In summary, this study systematically investigated the activation effects of three industrial solid waste activators—desulfurization gypsum (DG), carbide slag (CS), and steel slag (SS)—on red mud-based backfill materials, elucidating their mechanical properties, microstructural characteristics, and hydration mechanisms. The results demonstrate that a 6% dosage of these activators (DG/CS/SS) achieves the optimal activation of the pozzolanic activity in red mud. By utilizing solid wastes to replace high-cost chemical reagents, we successfully developed a mechanically and environmentally superior material suitable for mine goaf backfilling.

At the same time, DG, CS, and SS have different effects on the red mud-based filling materials. DG effectively improves the compressive strength of the materials but also makes the material brittle, which does not effectively improve the stress in the filling area and greatly reduces the effect in practical applications. Although CS does not significantly improve the compressive strength, it effectively improves the elastoplasticity of the material, which gives the material a good buffer effect and greatly improves the potential for using the material for goaf filling. In follow-up studies, mixing DG and CS with three dosage gradients of 6%, 7%, and 8% should be considered to study the effects of DG and CS on the pozzolanic activity of red mud and the physical and chemical properties of red mud-based filling materials so as to comprehensively improve the physical and chemical properties of red mud-based materials. In terms of the utilization of steel slag, we can consider extending the curing time and studying the effects of steel slag on the pozzolanic activity of red mud for a longer duration.

## Figures and Tables

**Table 1:** Composition table of raw materials used in experiment.

Components (Wt%)	Fe_2_O_3_	Al_2_O_3_	SiO_2_	Na_2_O	TiO_2_	CaO	SO_3_	P_2_O_5_	K_2_O
RM	49.84	19.33	12.20	8.53	7.93	0.87	0.55	0.19	0.16
FA	8.60	28.15	38.79	0.24	1.84	17.16	3.02	1.06	1.14
DG	0.00	0.03	0.04	0.06	0.00	49.19	50.64	0.00	0.00
CS	1.45	3.93	3.53	0.00	0.00	88.75	1.51	0.83	0.00
SS	31.83	6.23	21.20	3.26	1.79	25.66	3.68	2.13	4.22
PC	3.35	4.22	16.15	0.21	0.42	68.77	3.41	0.08	0.67

**Table 2:** Proportion design of red mud-based solid waste gel material (wt.%).

Sample	RM	FA	DG	CS	SS	PC
RFC	70	20	-	-	-	10
RFDC4	67.2	19.2	4	-	-	9.6
RFDC6	65.8	18.8	6	-	-	9.4
RFDC8	64.4	18.4	8	-	-	9.2
RFCC4	67.2	19.2	-	4	-	9.6
RFCC6	65.8	18.8	-	6	-	9.4
RFCC8	64.4	18.4	-	8	-	9.2
RFSC4	67.2	19.2	-	-	4	9.6
RFSC6	65.8	18.8	-	-	6	9.4
RFSC8	64.4	18.4	-	-	8	9.2

**Table 3:** Compressive strength and increment change in each specimen.

Sample	Compressive Strength/MPa					
	3 d	Rangeability/%	7 d	Rangeability/%	28 d	Rangeability/%
RFC	2.65	-	2.73	-	3.72	-
RFDC4	3.76	41.9%	4.66	70.7%	6.82	83.3%
RFDC6	4.09	54.3%	6.36	133.0%	7.36	97.8%
RFDC8	3.48	31.3%	4.38	60.4%	5.62	51.1%
RFCC4	2.56	−3.4%	2.98	9.2%	3.14	−15.6%
RFCC6	3.66	38.11%	4.00	46.5%	4.32	16.1%
RFCC8	3.07	15.85%	3.24	18.7%	3.63	−2.4%
RFSC4	2.33	−12.1%	2.52	−7.7%	3.26	−12.4%
RFSC6	2.45	−7.5%	2.67	−2.19%	3.35	−9.9%
RFSC8	1.81	−31.7%	2.36	−13.6%	3.10	−16.7%

**Table 4:** BET test results of various specimens.

Sample	RFC	RFDC6	RFCC6	RFSC6
Specific surface area (m^2^/g)	21.8451	23.8856	21.6598	21.9245
Total pore volume (cm^3^/g)	0.099563	0.125435	0.119396	0.111114
Average pore size (nm)	17.5122	18.6526	19.6011	19.4901

**Table 5:** Results of leachate testing.

Sample	Leaching Ion (mg/L)						
	As	Pb	Cd	Cr	Cu	Mn	Hg
RFC	0.0173	0.0066	<0.0012	0.0817	0.069.0	<0.0036	0.0005
RFDC6	0.0162	<0.0042	<0.0012	0.0691	0.022.6	<0.0036	0.0002
RFCC6	0.0157	<0.0042	<0.0012	0.0591	0.037.9	<0.0036	0.0003
RFSC6	0.0169	0.0057	<0.0012	0.0535	0.010.2	<0.0036	0.0001
GB8978-1996 limits	0.5	0.1	1.5	1.0	2.0	0.5	0.05

## Data Availability

The original contributions presented in the study are included in the article, further inquiries can be directed to the corresponding author.
